# Pharmacokinetics/pharmacodynamics of ceftazidime-avibactam in critically ill adult patients receiving continuous renal replacement therapy

**DOI:** 10.1128/aac.01438-25

**Published:** 2025-12-23

**Authors:** Chenyang Li, Yi Wang, Feng Chen, Lixuan Huang, Jianhua Dong, Wenjing Fan, Huijie Yue, Yongchun Ge

**Affiliations:** 1 National Clinical Research Center of Kidney Diseases, Jinling Hospital Affiliated to Nanjing University of Traditional Chinese Medicine 12581, Nanjing, Jiangsu, China; 2 Jinling Hospital Affiliated to Nanjing Medical University 12461, Nanjing, Jiangsu, China; 3 National Clinical Research Center of Kidney Diseases, Jinling Hospital Affiliated Hospital of Medical school, Nanjing University 12581, Nanjing, Jiangsu, China; 4 Department of Clinical Pharmacy, Jinling Hospital Affiliated Hospital of Medical school, Nanjing University 12581, Nanjing, Jiangsu, China; Providence Portland Medical Center, Portland, Oregon, USA

**Keywords:** ceftazidime-avibactam, CRRT, pharmacokinetics/pharmacodynamics, critically ill patients

## Abstract

Ceftazidime-avibactam (CAZ-AVI), a novel antibiotic, is effective in treating infections caused by carbapenem-resistant Gram-negative bacteria. However, in patients receiving continuous renal replacement therapy (CRRT), both the pharmacokinetics (PK) and pharmacodynamics (PD) of the drugs can be significantly altered. Currently, there remains a lack of clear guidelines regarding optimal dosing regimens for CAZ-AVI during CRRT. Prospectively, this study evaluated the PK/PD of CAZ-AVI in 21 critically ill patients receiving CRRT. We collected blood samples at 5–7 sampling points within one administration cycle and then determined the total plasma drug concentrations. Phoenix was used to calculate the PK parameters. The clearance at steady state (CL_SS_) of patients receiving CRRT was significantly reduced, and drug exposure was also significantly increased compared to healthy subjects. Notably, four patients demonstrated the free minimum plasma concentrations (fC_min_) of CAZ exceeding eight times the MIC, and 90.48% (19 cases) of the patients exhibited CAZ plasma concentrations exceeding the neurotoxicity threshold of 104 mg/L. PK/PD analysis indicated that the standard dosing regimen of 2.5 g every 8 hours of CAZ-AVI may pose a risk of excessive drug exposure. In addition, CRRT was the primary elimination pathway for CAZ-AVI in critically ill patients with acute kidney injury receiving CRRT. Significant differences in extracorporeal clearance were observed between continuous veno-venous hemodialysis (CVVHD) and continuous veno-venous hemofiltration (CVVH) for both CAZ and AVI; CVVH demonstrated higher clearance for CAZ and AVI compared to CVVHD. To prevent potential toxic reactions, it is urgent to establish a safer and more rational dosing regimen for patients receiving CRRT.

## INTRODUCTION

The emergence of drug-resistant Gram-negative bacterial infections has become a major public health concern, which is one of the leading causes of mortality in critically ill patients (1). The novel β-lactam (βL)/β-lactamase inhibitor combination—ceftazidime-avibactam (CAZ-AVI) has been demonstrated in clinical efficacy for carbapenem-resistant Gram-negative bacterial infections (2).

CAZ-AVI is primarily excreted unchanged via the kidneys. Consequently, patients with acute kidney injury or chronic kidney disease require dose adjustments based on their renal function. However, Wenzler et al. (3) reported that renal dose adjustment of CAZ-AVI may be associated with an increased risk of microbiological failure in critically ill patients receiving continuous renal replacement therapy (CRRT). Shields et al. also proposed there is a higher risk of clinical and microbiological failure with CAZ-AVI during CRRT (4).

Factors associated with CRRT may substantially affect the pharmacokinetics/pharmacodynamics (PK/PD) of antibiotics (5), and the application of CRRT could make the dosing regimen extremely challenging. Up to date, the PK/PD studies of CAZ-AVI during CRRT were limited, with existing evidence largely derived from studies with small sample sizes or case reports (3, 6–9). Notably, no PK/PD studies or extracorporeal clearance investigation have been conducted, specifically during continuous veno-venous hemofiltration (CVVH) or continuous veno-venous hemodialysis (CVVHD), apart from case reports. Moreover, recent research advocates for establishing both minimum and maximum limits in βL dosing to ensure therapeutic efficacy and to minimize toxicity (10, 11). Given that current research in this area has only examined whether the concentration of CAZ-AVI has reached the lower limit, in-depth investigations are required among patients receiving CRRT.

The present study was designed to investigate the PK/PD of CAZ-AVI in critically ill adult patients receiving CRRT, as well as the clearance of CAZ-AVI by CRRT.

## MATERIALS AND METHODS

### Study subjects

Patients admitted to the intensive care unit of the Jinling Hospital in Nanjing, China, between September 2024 and July 2025 were eligible for inclusion in the study if they met the following criteria: patient is over 18 years; receiving CAZ-AVI as part of standard care due to possible or microbiologically confirmed infection with extensively drug-resistant Gram-negative bacteria; and both CAZ-AVI and CRRT have been administered continuously for more than two consecutive days. Exclusion criteria included the following: patients allergic to CAZ-AVI and expected lifespan less than 72 hours.

### Clinical data collection

The demographic data of each patient, including age, gender, body weight, clinical diagnosis, pathogenic microorganisms, and Acute Physiology and Chronic Health Evaluation II (APACHE II) scores, were recorded. Based on 24-hour urine volume, patients were categorized into three groups: anuric group (<100 mL/24 hours), oliguric group (<400 mL/24 hours), and normal urine output group (400–2,000 mL/24 hours), with residual renal function estimated using 24-hour urine volume (12, 13). Microbial testing results included bacterial culture, antibiotic susceptibility test, and microbiological treatment outcomes. The Kirby-Bauer (KB) disk diffusion method was employed for antibiotic susceptibility testing. Clinical success was defined as survival without recurrence within 30 days of infection onset, resolution of infection symptoms, and no microbiologic failure. Patient outcomes were assessed by at least two independent investigators (C.Y. Li, Y. Wang, and F. Chen), and disagreements were resolved by a third. Microbiologic failure was the isolation of the same species after 7 days of CAZ-AVI treatment (4). Biochemical parameters, including serum creatinine (SCr), albumin, hematocrit (HCT), alanine aminotransferase, and aspartate aminotransferase, were recorded. Prescription details of CAZ-AVI, including the dosage, administration route, infusion duration, and treatment duration, were recorded. CRRT parameters, including modality, type of filter, blood flow rate, dialysate/replacement fluid flow rate, total effluent flow rate, and ultrafiltration rate, were also recorded.

For sample collection, all patients received intravenous infusion of 2.5 g of CAZ-AVI (comprising CAZ 2 g and AVI 0.5 g) every 8 hours as prescribed, initiated at the beginning of treatment and maintained until the day of sample collection, the infusion duration of CAZ-AVI was 1 hour, 2 hours, or 3 hours according to the prescription. For patients with a 1-hour infusion, blood samples were collected at 0, 1, 2, 3, 4, 6, and 8 hours; for 2-hour infusions, samples were collected at 0, 2, 3, 4, 6, and 8 hours; and for 3-hour infusions, samples were collected at 0, 3, 4, 6, and 8 hours. Blood samples were collected from the pre- and post-filter ports (pre-filter ports, a port on the intravenous tubing connecting the patient to the CRRT machine; post-filter ports, a port on the blood return line) of the hemofilter into EDTA-K2 anticoagulant tubes.

For determination of plasma drug concentration, blood samples were centrifuged at 2,125 × *g* for 10 minutes to separate the supernatant, which was then mixed with an equal volume of stabilizer (a mixed solution of 3-morpholinopropanesulfonic acid and ethylene glycol) and stored at −80°C until analysis. The total plasma concentrations of CAZ and AVI were quantified by validated liquid chromatography-mass spectrometry (14).

### PK/PD analysis of CAZ-AVI

Individual PK parameters for CAZ and AVI were estimated by total plasma concentrations from the pre-filter ports using non-compartmental analysis (NCA). NCA was performed using Phoenix WinNonlin (Version 8.1, Certara, Princeton, NJ, USA). Area under the concentration-time curve for a dosing interval (AUC_tau_) was determined by the linear trapezoidal rule. In addition, calculating the PK parameters of patients receiving 2.5 g q8h CAZ-AVI infusion for 2 hours and comparing them with those of healthy subjects under the same administration conditions (15) to explore the PK changes of CAZ and AVI in patients receiving CRRT.

The percentage of time with CAZ unbound concentrations above the minimum inhibitory concentration (fT >MIC) at 8 mg/L was considered the breakpoint for CAZ-AVI (16). The percentage of time with AVI unbound concentrations above the threshold concentration of 4 mg/L (fT >C_T_) was selected as PD parameter of CAZ and AVI efficacy, respectively (16). Simultaneously, achieving 100% fT >4 × MIC for CAZ and 100% fT >C_T_ for AVI is considered to represent attainment of the optimal PK/PD target (17). Due to the potential neurotoxicity associated with CAZ, we evaluated whether the free minimum plasma concentrations of CAZ (fC_min_) exceeded 8 × MIC (18). We also referred to the approach of Cojutti et al., which used 104 mg/L as the neurotoxicity threshold of CAZ to evaluate the risk of neurotoxicity in patients (19). The plasma protein binding for CAZ and AVI was reported as 10% and 7%, respectively (20), and unbound concentrations of CAZ and AVI were estimated by multiplying the total plasma concentrations by 0.90 and 0.93, respectively.

### Extracorporeal clearance by CRRT

Drug clearance by CRRT (CL_CRRT_) was calculated using the formula described previously (21); additionally, the contribution of CL_CRRT_ to the steady-state drug clearance (CL_SS_) was assessed by the formula (CL_CRRT_/CL_SS_) × 100%.


(1) $$CL_{CVVHD}=\left[QBI\times (1-HCT)\times CBI-QBO\times CBO\right]/CBI$$

(2) $$QBO=QBI\times (1-HCT)-Q_{Uf}$$

(3) $$CL_{CVVH}=\left[QBI\times (1-HCT)\times CBI-QBO\;\times \;CBO\right]/CBI$$

(4) $$QBO=QBI\times \left(1-HCT\right)-Q_{uf}-Q_{R}$$

CL_CVVHD_: drug clearance by CVVHD; CL_CVVH_: drug clearance by CVVH; QBI: pre-filter blood flow rate; HCT: hematocrit; CBI: plasma drug concentration at the port of pre-filter; QBO: post-filter blood flow rate; CBO: plasma drug concentration at the port of post-filter; Q_Uf_: ultrafiltration rate; Q_R_: replacement fluid flow rate.

### Statistical methods

SPSS 27.0 statistical software was used for analysis. Normally distributed continuous data were expressed as mean ± standard deviation, non-normally distributed continuous variables were reported as median with interquartile range (IQR), whereas categorical variables were expressed as count and percentage. PK parameters that conform to the lognormal distribution are presented as geometric mean (coefficient of variation%); in addition, geometric mean ratios (GMRs) and a 90% confidence interval (CI) were also shown; conversely, it is expressed as the median with IQR. Comparative analysis of continuous variables was performed using the independent samples t-test for normally distributed data and the Mann-Whitney U test for non-normally distributed data. *P*-values < 0.05 were considered statistically significant for all inter-group comparisons.

## RESULTS

### General information

A total of 238 blood samples were obtained from 21 critically ill patients receiving CRRT. The demographic and clinical characteristics of the included patients are summarized in Table 1. All patients received CRRT for more than 2 consecutive days, and the median (IQR) duration of continuous administration of 2.5 g q8h CAZ-AVI was 7 (5–17) days. Of these patients, infusion durations were distributed as follows: 3 completed within 1 hour, 8 within 2 hours, and 10 within 3 hours. The hemofilter applied during CRRT is a Nipro UT1100 filter (Nipro Corporation, Japan, with hollow fiber material being cellulose triacetate membrane). The blood flow rate was maintained at 160 mL/min, and the replacement fluid or dialysate flow rate was set at 2 L/h. The total effluent flow rate ranged from 20.42 to 42.03 mL/kg/h. Of all patients, 6 patients received post-dilution CVVH, and 15 patients received CVVHD.

**TABLE 1:** Demographics and clinical characteristics of critically ill patients^*a*^ receiving CRRT treated with CAZ-AVI^*c*^

Characteristics	All patients (*N* = 21)
Age (years)	47.19 ± 11.96
Gender	
Male, *n* (%)	15 (71.43)
Female, *n* (%)	6 (28.57)
Weight (kg)	72 [65.25, 85.50]
Clinical diagnosis	
Acute severe pancreatitis, *n* (%)	17 (80.95)
Sepsis, *n* (%)	3 (14.29)
Acute pancreatitis, *n* (%)	1 (4.76)
Infection sites	
Intra-abdominal infection, *n* (%)	13 (61.90)
Pulmonary infection, *n* (%)	3 (14.29)
Intra-abdominal infection and pulmonary infection, *n* (%)	5 (23.81)
Pathogens	
*Pseudomonas aeruginosa*, *n* (%)	8 (38.10)
*Klebsiella pneumoniae*, *n* (%)	6 (28.57)
*Klebsiella pneumoniae* combined with *Pseudomonas aeruginosa*, *n* (%)	4 (19.05)
*Acinetobacter baumannii* complex, *n* (%)	3 (14.28)
SOFA score^*a*^	15 (13,16)
APACHE II score^*a*^	
>15 points, *n* (%)	12 (57.14)
≤15 points, *n* (%)	9 (42.86)
24-Hour urine volume	
≤100 mL, *n* (%)	13 (61.90)
100–400 mL, *n* (%)	4 (19.05)
≥400 mL, ≤2,000 mL, *n* (%)	4 (19.05)
SCr (umol/L)^*b*^	78.40 [57.00, 157.35]
Serum albumin (g/L)	28.5 [26.80, 32.40]
Hematocrit value (L/L)	0.28 [0.26, 0.36]
Alanine aminotransferase (U/L)	24 [18, 45]
Aspartate aminotransferase (U/L)	43 [25.00, 73.50]

^*a*^ The SOFA score and APACHE II score were the patient’s scores on the day of sampling.

^*b*^ The patients were all undergoing CRRT, and the value of SCr was underestimated.

^*c*^ Data are presented as mean ± standard deviation, median [interquartile range], or *n* (%) of patients.

### PK/PD analysis

**Fig 1:**
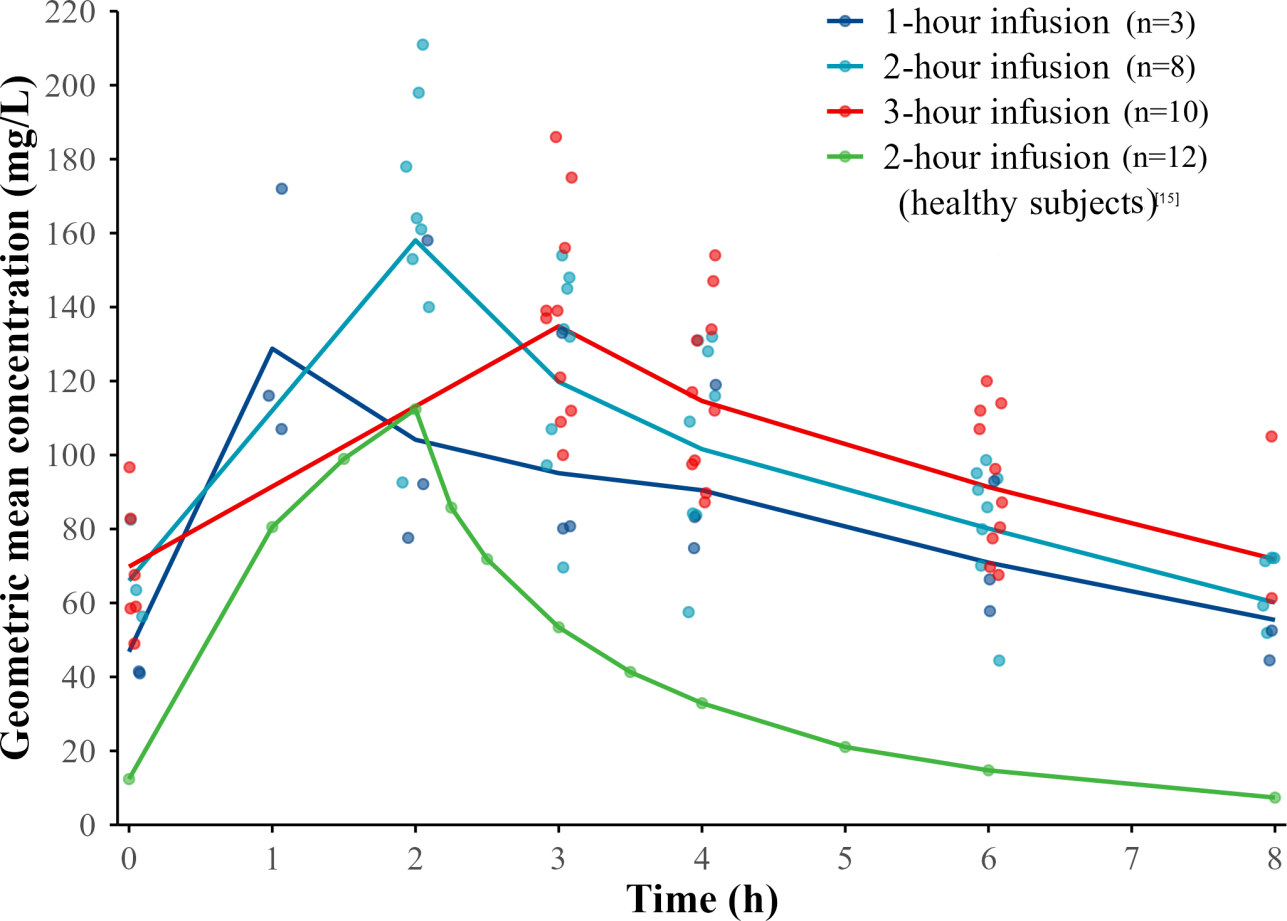
Geometric mean plasma concentration of ceftazidime vs time profiles by infusion duration at steady state. The profile of healthy subjects receiving CAZ-AVI treatment for 9 days is from reference 15. It was obtained by processing the original images using WebPlotDigitizer for trend comparison, rather than for direct numerical comparison.

**Fig 2:**
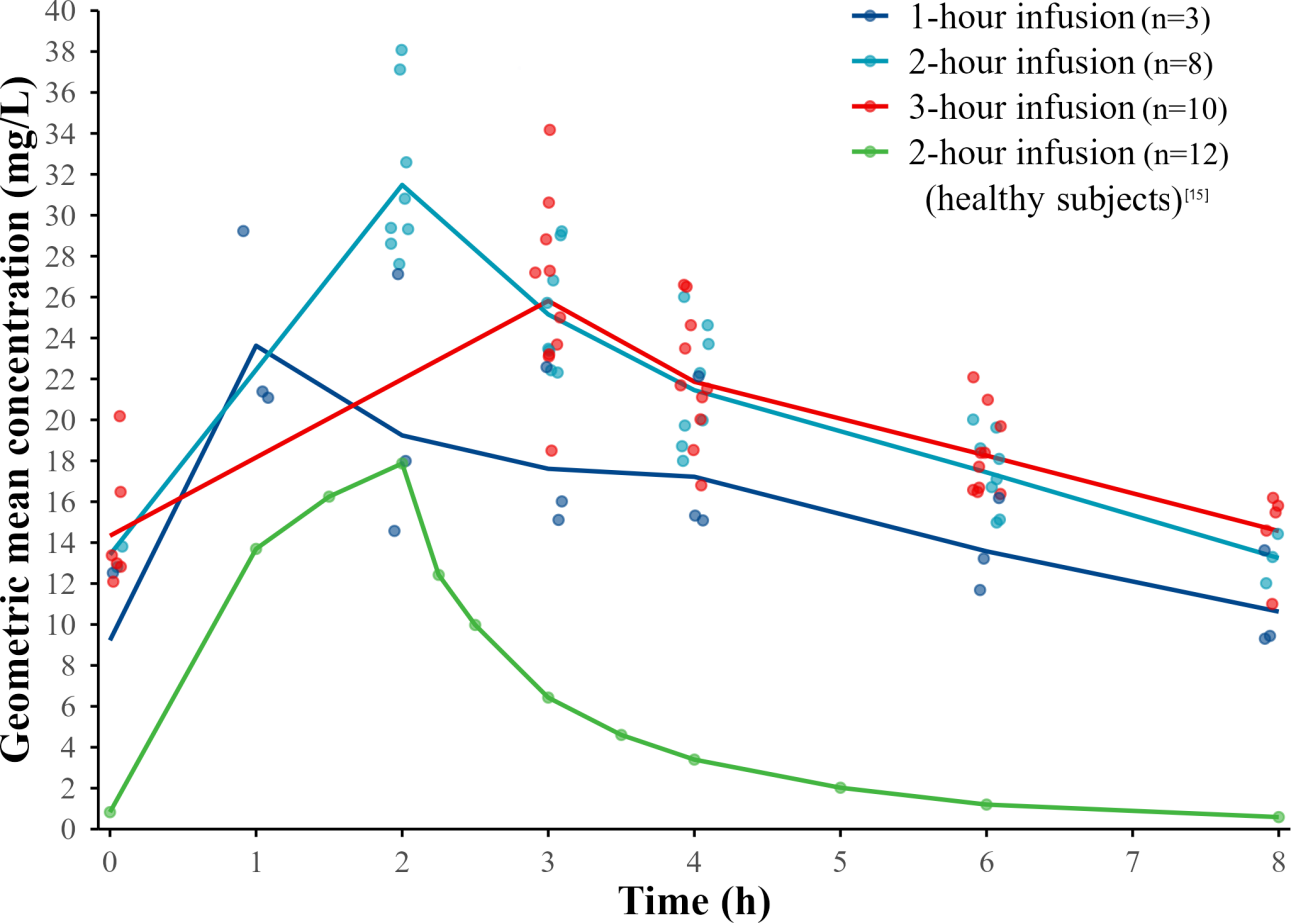
Geometric mean plasma concentration of avibatam vs time profiles by infusion duration at steady state. The profile of healthy subjects receiving CAZ-AVI treatment for 9 days is from reference 15. It was obtained by processing the original images using WebPlotDigitizer for trend comparison, rather than for direct numerical comparison.

According to the concentration-time curve, the AUC of CAZ-AVI in patients receiving CRRT was significantly higher than that in healthy subjects (Fig. 1 and 2).

Compared with the PK parameters of CAZ-AVI in healthy subjects (15), under the same administration conditions of CAZ-AVI 2.5 g q8h via a 2-hour infusion, CL_SS_ of CAZ and AVI in patients receiving CRRT decreased by approximately 60% and 74%, respectively. Meanwhile, the AUC_tau_ increased by approximately 1.48-fold and 2.82-fold, the maximum plasma concentration (C_max_) increased by approximately 0.42-fold and 0.79-fold, and the half-life (t_1/2_) was prolonged by approximately 3 hours (Table 2).

**TABLE 2:** Comparative PK parameters of CAZ-AVI among patients receiving CRRT and healthy subjects^*c*^^,^^*d*^

PK parameters	Ceftazidime		Avibactam	
	Patients undergoing CRRT (*n* = 8)^*a*^	Healthy subjects (*n* = 12)^*b*^	Patients undergoing CRRT (*n* = 8)^*a*^	Healthy subjects (*n* = 12)^*b*^
C_max_ (mg/L)	158.06 (22.52)	111.1 (14.6)	31.48 (12.46)	17.6 (18.1)
C_min_ (mg/L)	56.71 (20.81)	ND	12.07 (17.23)	ND
AUC_tau_ (h·mg/L)	796.96 (21.36)	321.5 (15.5)	166.50 (11.10)	43.6 (19.1)
t_1/2_ (h)	5.35 (30.67)	2.5 (13.0)	5.89 (27.35)	2.8 (31.3)
V_SS_ (L)	18.42 (47.40)	ND	24.78 (35.41)	ND
CL_SS_ (L/h)	2.50 (27.86)	6.2 (15.5)	3.00 (10.54)	11.5 (19.1)

^*a*^ Data are derived from the results of this study. Patients undergoing CRRT received a 2.5 g (CAZ 2,000 mg + AVI 500 mg) infused for 2 hours every 8 hours.

^*b*^ Data are from reference 15. Healthy subjects received a 2.5 g (CAZ 2,000 mg + AVI 500 mg) infused for 2 hours every 8 hours for 9 days.

^*c*^ Data are presented as geometric mean (coefficient of variation%).

^*d*^ CRRT: continuous renal replacement therapy; C_max_: maximum plasma concentration; C_min_: minimum plasma concentration; AUC_tau_: area under the concentration-time curve for a dosing interval; t_1/2_: half-life; V_SS_: volume of distribution at steady state; CL_SS_: clearance at steady state; ND: no data.

Significant differences were observed in C_max_ and volume of distribution at steady state (V_SS_) of both CAZ and AVI between the CVVH and CVVHD. Specifically, the V_SS_ of CAZ and AVI was higher in patients receiving CVVH (*P* = 0.02 and *P* = 0.007, respectively), while the C_max_ of CAZ and AVI was higher in patients receiving CVVHD (*P* = 0.02 and *P* = 0.026, respectively) (Table 3). In addition, there is a difference in t_1/2_ of AVI between CVVH and CVVHD (*P* = 0.012), the t_1/2_ of AVI was higher in patients receiving CVVH (Table 3).

**TABLE 3:** Comparative analysis of PK parameters of CAZ-AVI in patients receiving CVVH and CVVHD*^a^*^,^*^b^*^,^*^c^*^,^*^d^*

PK parameters	Ceftazidime					Avibactam				
	CVVHD (*n* = 15)	CVVH (*n* = 6)	GMR	90% CI	*P*-value	CVVHD (*n* = 15)	CVVH (*n* = 6)	GMR	90% CI	*P*-value
C_max_ (mg/L)	153.26 (20.50)	118.22 (18.24)	1.30	(1.09–1.54)	0.02*	29.09 (16.56)	23.88 (15.05)	1.22	(1.06–1.40)	0.026*
C_min_ (mg/L)	60.67 (27.95)	55.56 (17.94)	1.09	(0.88–1.35)	0.421	12.24 (22.64)	12.65 (9.40)	0.97	(0.81–1.16)	0.914
AUC_tau_ (h·mg/L)	829.99 (19.46)	675.12 (20.68)	1.23	(1.03–1.46)	0.054	161.43 (15.58)	143.94 (7.60)	1.12	(0.99–1.27)	0.095
t_1/2_ (h)	5.42 (21.97)	6.41 (13.33)	0.85	(0.71–1.00)	0.114	5.92 (21.11)	7.59 (11.34)	0.78	(0.66–0.92)	0.012*
V_SS_ (L)	18.62 (33.59)	27.06 (23.14)	0.69	(0.52–0.90)	0.02*	26.14 (29.96)	37.71 (7.43)	0.69	(0.59–0.81)	0.007**
CL_SS_ (L/h)	2.41 (21.23)	2.97 (22.26)	0.81	(0.68–0.96)	0.119	3.04 (2.81–3.31)	3.39 (3.29–3.55)			0.213

^*a*^ Data are presented as geometric mean (coefficient of variation%), except CL_SS _of avibactam, which is presented as median (interquartile range). Comparative analyses are presented as GMRs of CVVHD and CVVH with a 90% CI and the independent samples t-test *P*-values.

^*b*^ CVVH: continuous veno-venous hemofiltration. CVVHD: continuous veno-venous hemodialysis.

^*c*^ C_max_: maximum plasma concentration; C_min_: minimum plasma concentration; AUC_tau_: area under the concentration-time curve for a dosing interval; t_1/2_: half-life; V_SS_: volume of distribution at steady state; CL_SS_: clearance at steady state.

^*d*^ *P < 0.05, **P < 0.01.

Among the seven patients whose isolated strains are sensitive to CAZ-AVI, the lowest C_min_ of CAZ and AVI were 37.60 mg/L and 11.10 mg/L, respectively. Consequently, these patients achieved the joint PK/PD target. Among all the patients, the lowest C_min_ of CAZ and AVI were 37.60 mg/L and 7.89 mg/L, the fC_min_ of four patients exceeded 8 × MIC, with corresponding C_min_ of 72.20 mg/L, 72.10 mg/L, 89.50 mg/L, and 105.00 mg/L, respectively. However, no apparent adverse or neurotoxic reactions were observed among the patients enrolled in the study.

### Clearance of CAZ-AVI by CRRT

**Fig 3:**
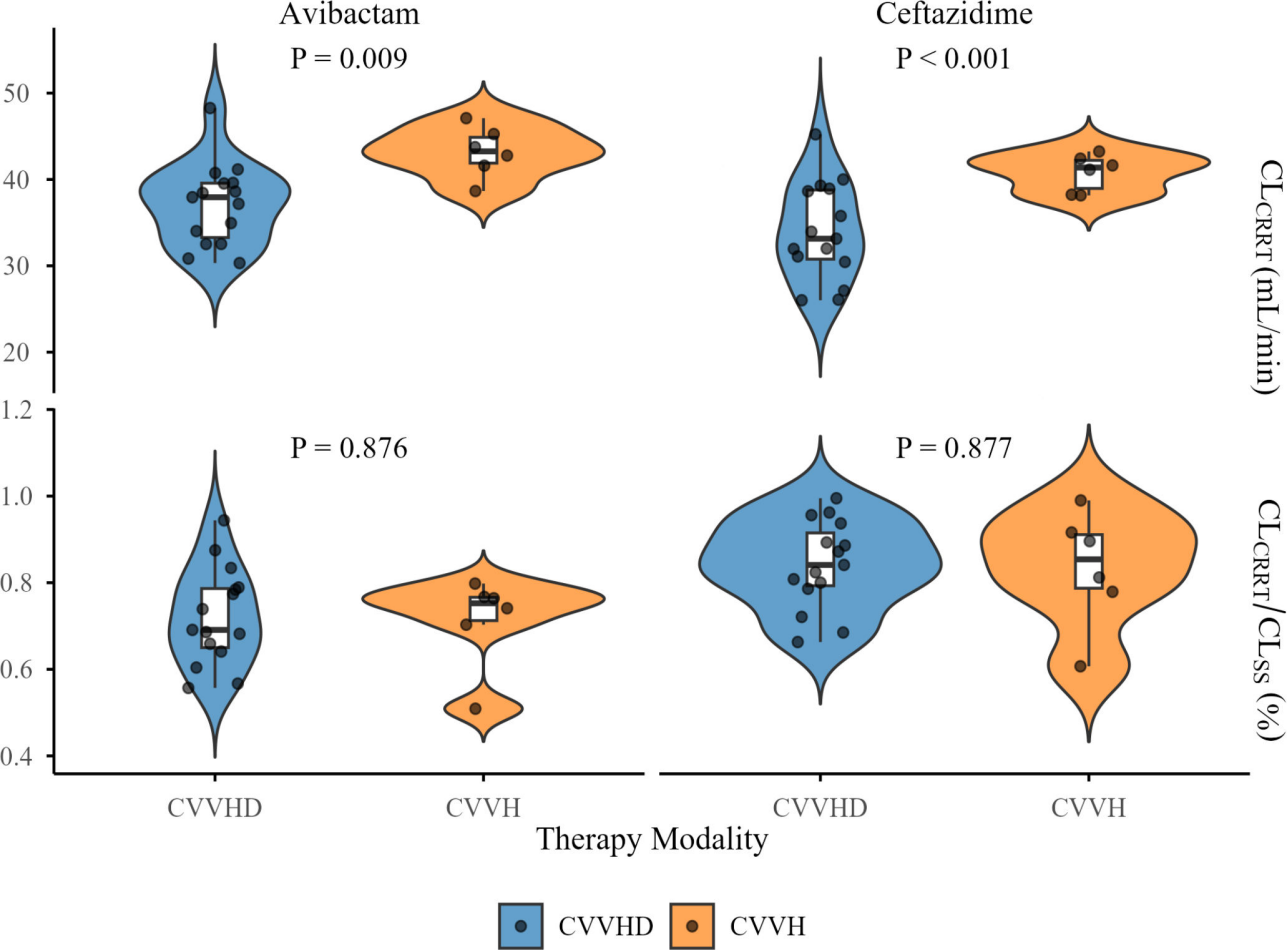
The clearance of CAZ-AVI by CRRT (CL_CRRT_) and the proportion of CL_CRRT_ in the CL_ss_ under the CVVH and CVVHD modalities.

Statistically significant differences were observed in the clearance of both CAZ and AVI by CVVH and CVVHD (*P* < 0.001 and *P* = 0.009, respectively), with CVVH demonstrating higher clearance for both agents. However, no significant differences were found in the ratio of CL_CRRT_ to CL_SS_ for either drug between the two modalities (*P* = 0.877 and *P* = 0.876, respectively) (Fig. 3).

### Microbial treatment outcomes

The majority of the patients (85.71%) were infected with *Klebsiella pneumoniae* (KP) and/or *Pseudomonas aeruginosa* (PA), except for three patients who were infected with *Acinetobacter baumannii* complex (ABC). All the KP isolated from the patients were carbapenem-resistant Enterobacteriaceae (CRE) bacteria, and all the PA strains isolated were difficult-to-treat (DTR)-resistant PA. PA was identified in 12 cases, all of which were resistant to CAZ-AVI, antimicrobial treatment failed in all cases, except for cases with no available data. KP was identified in 10 cases, and 7 cases were infected with strains sensitive to CAZ-AVI. All the seven patients achieved the joint PK/PD target and completed pathogen clearance. In contrast, three cases were infected with KP strains that were resistant to CAZ-AVI, and antibacterial treatment succeeded in one case and failed in two cases (Table 4).

**TABLE 4:** Achievement of joint PK/PD targets in patients, along with antibiotic susceptibility test and microbial treatment outcomes*^a^*^,^*^b^*

ID	Infection site	Pathogenic microorganisms	KB value (breakpoint ≤20, ≥21)	Susceptibility	Pathogen clearance	Clinical outcome
1	IAI	KP	23	S	Succeed	Succeed
2	IAI	KP	22	S	Succeed	Succeed
3	IAI	PA	6	R	Failed	Failed
4	IAI	KP/PA	10/6	R/R	Succeed/failed	Succeed/failed
5	PI	ABC	ND	ND	ND	ND
6	IAI/PI	KP	16	R	Failed	Failed
7	PI	KP/PA	25/6	S/R	Succeed/failed	ND
8	IAI/PI	ABC	ND	ND	ND	ND
9	IAI/PI	KP	23	S	Succeed	Succeed
10	IAI/PI	PA	6	R	Failed	Failed
11	IAI	PA	6	R	Failed	Failed
12	IAI	ABC	ND	ND	ND	ND
13	IAI/PI	KP	22	S	Succeed	Succeed
14	IAI	PA	6	R	Failed	Failed
15	IAI	KP/PA	18/6	R/R	Succeed/succeed	Succeed/succeed
16	IAI	PA	6	R	Failed	Failed
17	IAI	KP	23	S	ND	ND
18	IAI	KP/PA	22/6	S/R	Succeed/failed	Succeed/failed
19	IAI	PA	6	R	Failed	Failed
20	IAI	PA	6	R	Failed	Failed
21	PI	PA	6	R	Failed	Failed

^*a*^ IAI: intra-abdominal infection; PI: pulmonary infection; KP: *Klebsiella pneumoniae*; PA: *Pseudomonas aeruginosa*; ABC: *Acinetobacter baumannii* complex; S: susceptible; R: resistant; ND: no data, the lack of microbiological treatment and clinical outcomes can be attributed to patients being transferred to other hospitals and lost to follow-up within 1 week after initiating therapy, or to their switching to alternative antibiotics during the course of treatment.

^*b*^ The KB disk diffusion method was employed for antibiotic susceptibility testing. Clinical success was defined as survival without recurrence within 30 days of infection onset, resolution of infection symptoms, and no microbiologic failure. Patient outcomes were assessed by at least two independent investigators (C.Y. Li, Y. Wang, and F. Chen), and disagreements were resolved by a third. Microbiologic failure was the isolation of the same species after 7 days of CAZ-AVI treatment (4).

## DISCUSSION

This study conducted an in-depth analysis of the PK/PD of CAZ and AVI in patients receiving CRRT, as well as the clearance of CAZ-AVI by CRRT. We set the upper limit for CAZ-AVI in patients receiving CRRT, and compared the clearance of CAZ-AVI by different CRRT modalities for the first time.

Our findings indicated that CL_SS_ of CAZ-AVI was reduced in critically ill patients receiving CRRT. This reduction may be attributed to the relatively lower solute clearance of CRRT compared to that of normal renal function. The decreased CL_SS_ led to a significantly increased AUC_tau_ and prolonged t_1/2_, which suggested a potential risk of drug accumulation at standard dosing regimens in this patient population. In addition, 4 patients exhibited fC_min_ >8 × MIC, 90.48% (19 cases) of the patients exhibited CAZ plasma concentrations exceeding the neurotoxicity threshold of 104 mg/L, which may pose a potential risk of neurotoxicity (10, 11, 18). This suggests that the dosage of CAZ-AVI may require adjustment in patients undergoing CRRT. Gatti et al. proposed a loading dose of 2.5 g over 2 hours, followed by a continuous infusion of 1.25 g every 8 hours could achieve the optimal PK/PD target and microbial eradication, which may be a valuable approach (8). The adoption of a continuous infusion strategy for CAZ-AVI during CRRT may be a potentially advantageous strategy for minimizing the risk of neurotoxicity and for administering lower daily dosing regimens.

Compared with previous studies on the PK/PD of CAZ-AVI in patients receiving CRRT, apart from case reports (3, 6, 7), two relevant studies were conducted in patients receiving continuous veno-venous hemodiafiltration (CVVHDF) (8, 9). In the study by Gatti et al. (8), median (IQR) CL_SS_ of CAZ and AVI were reported as 2.39 L/h (2.05–2.94 L/h) and 2.56 L/h (2.22–2.96 L/h), respectively. In the study by O'Jeanson et al. (9) reported median CL_SS_ and V_SS_ for CAZ as 4.54 L/h and 73.2 L, respectively, while for AVI, the median CL_SS_ and V_SS_ were 10.5 L/h and 102 L, respectively. The higher CL_SS_ and V_SS_ observed in O'Jeanson et al.’s study may be associated with variations in underlying diseases, pathophysiological conditions, and residual renal function of the patients included in their study. Specifically, the elevated CL_SS_ could be attributed to higher residual renal function, while the increased V_SS_ might be due to fluid resuscitation-induced expansion of total body fluid volume or the presence of severe edema (22).

The results of the present study demonstrated that the average clearance values of CAZ and AVI by CVVHD were 33.98 ± 5.62 mL/min and 35.53 ± 7.79 mL/min, respectively, while those by CVVH were 40.80 ± 2.14 mL/min and 43.19 ± 2.94 mL/min, respectively. For low-molecular-weight drugs, assuming a protein binding rate of 0%, the maximum possible clearance rate is considered to be equivalent to the total effluent flow rate (23). In our study, the CRRT total effluent flow rate ranged from 2,180 to 2,500 mL/h, which corresponds to a theoretical maximum drug clearance of approximately 36.33–41.67 mL/min. However, the clearance of CAZ and AVI by CRRT in the present study was higher than that in other studies and the theoretical maximum clearance. Wenzler et al. (3) reported mean clearance of CAZ and AVI by CVVH as 27.33 mL/min and 26.50 mL/min, respectively, while O'Jeanson et al. (9) reported median clearance of CAZ and AVI by CVVHDF as 14.5 mL/min and 8.33 mL/min, respectively. This may be attributed to the following factors: (i) The formula used to calculate clearance by CRRT exhibits variations across different studies: previous studies estimated clearance based on the sieving coefficient/saturation coefficient, which does not account for the adsorption capacity of the filter membrane. In contrast, we used a method based on plasma drug concentrations at pre- and post-filter ports (equations (1)–(4)-4), which incorporates both dialysis/filtration clearance and membrane adsorption. (ii) The sample size limitations: The study by Wenzler et al. (3) evaluated only a single patient, while O'Jeanson et al. (9) included only four patients. Such limited sample sizes may introduce potential bias and limit the generalizability of the findings. (iii) Variations in CRRT parameters: In Wenzler et al.’s (3) study, patients were treated with CVVH using a 1.6 m² polyethersulfone membrane filter, with fixed blood and ultrafiltrate flow rates of 200 mL/min and 2 L/h, respectively. In O'Jeanson et al.’s (9) study, patients received CVVHDF using AN69ST filter membranes and ST150 SET filters, with blood flow rates of 100–150 mL/min, dialysate flow rates of 1,000 mL/h, and ultrafiltrate flow rates of 1,000–1,500 mL/h, at a CVVHDF dose intensity of 25–30 mL/kg/h. Differences in CRRT settings, including membrane material, blood flow rate, dialysate/replacement fluid flow rate, and ultrafiltration rate, may significantly influence drug clearance. Additionally, we found that CVVH achieved significantly higher clearance for CAZ and AVI compared to CVVHD; however, no significant difference was observed in CL_SS_ between the two modes. This may be due to higher renal clearance in patients receiving CVVHD. Notably, among the four patients with normal urine output, three received CVVHD.

In this study, the contribution of CL_CVVH_ to CL_SS_ for CAZ and AVI was 0.83 ± 0.13 and 0.71 ± 0.11, respectively, while the contribution of CL_CVVHD_ to CL_SS_ for CAZ and AVI was 0.84 ± 0.10 and 0.72 ± 0.17, respectively. Wenzler et al. (3) observed only one anuric patient with renal failure, with blood and ultrafiltrate flow rates of 200 mL/min and 2 L/h, respectively. In that case, the contribution of CVVH to total clearance of CAZ and AVI was 57.1% and 54.3%, respectively. O'Jeanson et al. (9) reported median proportion of the clearance by CVVHDF in the total clearance for CAZ and AVI was 19.8% and 5.3%, respectively. The lower contribution rates observed in O'Jeanson et al.’s (9) study may be attributed to the relatively higher residual renal function of the patients. CAZ/AVI has relatively low molecular weights (547/265), exhibits low protein-binding rates (10%/7%), and is predominantly eliminated via renal excretion (90%/97%) (20). Within the cohort of patients included in this study, 17 individuals (80.95%) presented with anuria or oliguria, indicating minimal residual renal function available for the elimination of CAZ-AVI, CRRT emerged as the primary route of CAZ-AVI clearance. Consequently, the clearance of both CAZ and AVI through CRRT accounted for a significant proportion of the CL_SS_.

Nevertheless, this study also has some limitations: (i) The overall sample size was relatively small, with only six patients receiving CVVH. (ii) The free plasma drug concentrations of CAZ and AVI were estimated based on assumed free fractions, as only total plasma concentrations were measured. (iii) Patients were already undergoing CRRT at enrollment, with low SCr levels and mostly anuric or oliguric status, making accurate assessment of residual renal function challenging. Relying solely on urine output may not provide sufficient accuracy. (iv) Ultrafiltrate/dialysate samples were not collected to calculate sieving or saturation coefficients, which could have provided additional insights into drug clearance through the filter membrane.

### Conclusion

CAZ-AVI is extensively cleared by CRRT and represents a major route of drug elimination in critically ill patients receiving CRRT. CVVH demonstrates higher clearance for CAZ and AVI compared to CVVHD. However, compared with healthy subjects, the CL_SS_ of these patients was significantly lower, and drug exposure was markedly increased. Notably, fC_min_ may exceed 8 × MIC, and 90.48% (19 cases) of the patients exhibited CAZ plasma concentrations exceeding the neurotoxicity threshold of 104 mg/L under the dosing regimen of CAZ-AVI 2.5 g q8h. This situation may present a potential risk of neurotoxicity; it is urgent to establish a safer and more rational dosing regimen for patients receiving CRRT.

## Data Availability

All raw pharmacokinetic and pharmacodynamic data generated and analyzed during this study have been deposited in Dryad and are publicly available via the following DOI: https://doi.org/10.5061/dryad.fxpnvx16s.
