# Erratum for Li et al., "Pharmacokinetics/pharmacodynamics of ceftazidime-avibactam in critically ill adult patients receiving continuous renal replacement therapy"

**DOI:** 10.1128/aac.00438-26

**Published:** 2026-06-04

**Authors:** Chenyang Li, Yi Wang, Feng Chen, Lixuan Huang, Jianhua Dong, Wenjing Fan, Huijie Yue, Yongchun Ge

## ERRATUM

Volume 70, no. 2, e01438-25, 2025, http://doi.org/10.1128/aac.01438-25. Author Affiliations: The first affiliation should read “Jinling Clinical Medical College, Nanjing University of Chinese Medicine, Nanjing, Jiangsu, China.”

